# Effects of conflict trial proportion: A comparison of the Eriksen and Simon tasks

**DOI:** 10.3758/s13414-020-02164-2

**Published:** 2020-12-02

**Authors:** Karin M. Bausenhart, Rolf Ulrich, Jeff Miller

**Affiliations:** 1 Department of Psychology, University of Tübingen, Schleichstr. 4, 72076 Tübingen, Germany; 2 University of Otago, Dunedin, New Zealand

**Keywords:** Attention and executive control, Conflict tasks, Eriksen task, Simon task, Cognitive control, Congruency proportion, Gratton effect, Contingency learning

## Abstract

Two experiments examined global and local behavioral adaptation effects within and across the Eriksen task, where conflict is based on stimulus letter identities, and the Simon task, where conflict is based on stimulus and response locations. Trials of the two tasks were randomly intermixed, and the list-wide proportion of congruent trials was varied in both tasks (Experiment 1) or in just one task (Experiment 2). The global adaptation effect of list-wide congruency proportion (LWPC effect) was at least as large in the Simon task as in the Eriksen task. Likewise, the local adaptation effect of previous-trial congruency (Gratton effect) was at least as large in the Simon task as in the Eriksen task. In contrast to prior studies investigating transfer across Stroop and Simon tasks, there was no dissociation between global and local adaptation effects regarding their transfer across the different conflict tasks. In fact, both local and global adaptation effects appeared largely task-specific, because there was no or only little transfer of either Gratton effects or LWPC effects from the Eriksen to the Simon task or vice versa. On the whole, the results suggest that behavioral adaptation observed in the present design does not carry over from one of these tasks to the other, suggesting no involvement of a higher-order, task-general mechanism of cognitive control.

## Introduction

The concept of selective attention implies that people are capable of monitoring a certain source of information while suppressing irrelevant information that impinges on the perceptual system at the same time. Research on selective attention has demonstrated, however, that irrelevant information usually cannot be completely suppressed, but may interfere with the processing of relevant information (Pashler, 1998). This fact is especially evident in conflict tasks, in which participants are asked to process task-relevant information in the presence of potentially conflicting task-irrelevant information. Common examples of such tasks are the Eriksen flanker task (Eriksen & Eriksen, 1974), the Simon task (Simon, 1969), and the Stroop task (Stroop, 1935).

### Typical conflict tasks

For instance, in the Eriksen task, a centrally presented target letter is flanked by adjacent letters. Participants are asked to make a speeded choice to the target letter while ignoring the flanker letters. Responses are usually faster and more accurate when the target and flanker letters afford the same response (congruent condition) than when they afford different responses (incongruent condition). In the Simon task, participants also make a speeded choice to a target stimulus, which is, however, presented to the left or right of fixation. Even though the spatial location of the target is task-irrelevant, responses are typically faster when the location of the target coincides with the response side (congruent condition), than when target location is opposite to the side of the correct response (incongruent condition). Finally, in a typical Stroop task, participants have to name the print color of a color word while ignoring its semantic content. Nonetheless, responses are faster when the print color is congruent with its semantic content (e.g., the word BLUE printed in blue) rather than incongruent (e.g., the word BLUE printed in red). Superficially, the cognitive demands appear similar in all of these conflict tasks; namely, it is necessary to process task-relevant information while suppressing conflicting task-irrelevant information. This naturally raises questions about how we adapt our behavior in the presence of conflicting information and—central to the present study—whether the underlying mechanisms are the same or different between different conflict tasks.

### Behavioral adaptation in conflict tasks

Two basic observations have been particularly revealing about behavioral adaptation in such conflict tasks. First, congruency effects in a current trial are especially pronounced when this trial was preceded by a congruent trial. By contrast, congruency effects are reduced when the current trial was preceded by an incongruent trial. This so-called *Gratton* effect (e.g., Gratton et al., 1992, Stürmer et al., 2002) therefore reflects adaptive behavioral changes on a *local* (trial-by-trial) basis. Second, another example of behavioral adaptation emerges when the relative frequency of congruent and incongruent trials is varied between blocks of trials. Specifically, the proportion of congruent versus incongruent trials within an experimental block modulates the size of the congruency effect (Logan & Zbrodoff, 1979); its size is larger in blocks with a high compared to a low proportion of congruent trials. This modulation of the congruency effect, commonly termed the *list-wide proportion congruency* (LWPC) effect, may thus be described as a *global* (block-wide) adaptation effect (for an overview, see Bugg and & Crump, 2012).

Several accounts have been put forward to explain sequential (local) and list-wide (global) modulations in the magnitude of congruency effects. One class of theories may be subsumed as conflict-monitoring accounts. Accordingly, the Gratton effect is attributed to a reactive and relatively transient process that regulates processing of relevant and irrelevant information directly after a conflict (e.g., competing activation of different response alternatives) is detected (e.g., Botvinick et al., 2001, Gratton et al., 1992, Verguts & Notebaert, 2008, 2009). Similarly, the LWPC effect can also be conceived as an adaptive control process: The frequent experience of processing conflict in blocks with mostly incongruent trials, as opposed to mostly congruent trials, may trigger attentional control processes, for example an increase of attention to relevant and/or a decrease of attention to irrelevant stimulus features (e.g., Logan & Zbrodoff, 1979). Furthermore, one may even argue that the Gratton and the LWPC effect are simply two sides of the same coin, because in blocks with mostly congruent trials, any current trial is more likely to be preceded by a congruent trial than by an incongruent trial, thereby boosting the block-wide congruency effect on a trial-by-trial level. Analogously, in mostly incongruent blocks, any current trial is more likely preceded by an incongruent trial than by a congruent one, thereby reducing the block-wide congruency effect. Accordingly, the LWPC effect would emerge as a direct consequence of the trial-by-trial adaptations reflected in the Gratton effect (e.g.,Verguts & Notebaert, 2008). In any case, these accounts imply the existence of dedicated mechanisms that regulate stimulus processing through higher-order cognitive control.

A second class of explanations, the contingency-based accounts, do not depend on conflict detection, and therefore, do not posit the existence of a genuine cognitive control mechanism. Rather, these theories stress the role of lower-level processes as contingency learning or stimulus-specific priming. For example, sequential modulations of congruency effects may be explained through episodic memory processes such as feature-repetition priming or feature-binding, resulting in processing benefits for exact stimulus/response repetitions or complete stimulus/response alternations, as opposed to partial repetitions (e.g., Hommel et al., 2004, Mayr et al., 2003). Crucially, exact repetitions or complete alternations occur exclusively in congruent trials preceded by congruent ones and in incongruent trials preceded by incongruent ones. Thereby, especially fast responses in these trials will produce a typical Gratton effect. Moreover, when congruency proportion is varied across experimental blocks, behavioral adaptation may result from learning of stimulus-response contingencies. That is, the task-irrelevant features become predictive of the correct response, thereby enabling especially fast responses, simply because they are frequently paired with a specific task-relevant feature. For example, in a Stroop task, the word BLUE would be paired especially often with blue font in mostly congruent blocks (speeding responses in these congruent trials, resulting in a large congruency effect), but especially often with red font in mostly incongruent blocks (speeding responses in these incongruent trials, resulting in a reduced congruency effect, Schmidt & Besner, 2008; for overviews, see Schmidt 2013, 2019, Bugg & Crump 2012).

There is increasing evidence that behavioral adaptation effects like the LWPC and the Gratton effect may often (if not mostly) be better explained by low-level accounts as contingency learning rather than by genuine cognitive control processes (Schmidt, 2013, 2019). Nonetheless, these two classes of explanations are not necessarily mutually exclusive, and it has been demonstrated that both may play a role in behavioral adaptation effects (e.g., Akçay & Hazeltine, 2007, 2011, cf. Braem et al., 2019, for a set of recommendations on how to control for low-level confounds based on stimulus contingencies in order to isolate genuine cognitive control effects).

It should be noted that we do not aim to distinguish between these classes of accounts. Therefore, in the following, we refer to LWPC and Gratton effects as ‘behavioral adaptation’ effects, which is more neutral with respect to the underlying mechanisms than, for example, ‘conflict adaptation’. Nonetheless, the presence or absence of transfer of behavioral adaptation across different tasks (which is at the core of the present study) may be informative as to this distinction. On the one hand, as reviewed comprehensively in Braem et al., (2014), models of (genuine) cognitive control/conflict monitoring allow for a certain transfer of sequential effects across different tasks, for example, when the task-relevant information (Botvinick et al., 2001), the conflict type (Egner, 2008), or task set (Hazeltine et al., 2011) repeats across otherwise different tasks. For instance, if a cognitive control mechanism acts to strengthen processing of task-relevant information after a conflict is detected, this should facilitate processing of a subsequent stimulus with the same task-relevant information, whether it is accompanied by the same or a different type of task-irrelevant information. In other words, behavioral adaptation effects induced by such a higher-level control mechanism may transfer across different conflict types. On the other hand, lower-level, contingency-based accounts of behavioral adaptation naturally entail that the effects of congruency sequence are specific to the recently processed stimulus-response event. Thus, these accounts do not readily predict transfer of behavioral adaptation effects across different tasks, stimuli, or conflict types.

### Common or separate mechanisms underlying behavioral adaptation in different conflict tasks?

As outlined above, Gratton effects and LWPC effects may both be based on the same trial-by-trial behavioral adjustments. Moreover, both types of effects have been observed for all three conflict task types introduced above (for overviews, see Braem et al., 2014, Bugg and Crump, 2012). Therefore, it seems parsimonious to assume that a single mechanism underlies these behavioral adaptation effects in the different conflict tasks.

Some researchers, however, have suggested that the Gratton effect and the LWPC effect may reflect distinct mechanisms (Aben et al., 2017; De Pisapia & Braver, 2006; Dosenbach et al., 2008; Funes et al., 2010; Torres-Quesada et al., 2013). For example, De Pisapia and Braver (2006) have argued within their Dual Mechanisms of Control framework that two different conflict units operate upon the detection of conflict. Specifically, one control mechanism exerts a transient, reactive form of cognitive control, whereby task-irrelevant information is suppressed in response to conflict detection. A second control mechanism exerts more sustained, proactive control by priming pathways responsible for the processing of task-relevant information prior to stimulus onset.

Behavioral evidence consistent with such a distinction has been reported by Funes et al., (2010), who randomly intermixed trials which induced different conflict types. Specifically, in each trial of their experiment, participants responded with their left or right hand to the direction of an arrow that could point up or down. Because each arrow was presented at one of four locations (left, right, above, or below of fixation), two different types of conflict were created depending on the arrow’s location. When the arrow appeared to the left or right of fixation, its location could be congruent or incongruent with the response side, thereby creating a Simon conflict. When the arrow appeared above or below the central fixation point, its location could be either congruent or incongruent with the direction of the arrow, thereby creating a spatial Stroop conflict. Importantly, the proportion of congruent trials for the Simon task was varied between separate halves of the experiment, whereas this proportion was always kept constant at 50 % for the spatial Stroop task.

The authors then assessed the global and local influences on the congruency effect for both conflict types, depending on whether the conflict type was repeated or changed between subsequent trials. This analysis revealed a theoretically interesting dissociation between the Gratton and the LWPC effect. First, the congruency of the previous trial *n* − 1 affected the congruency effect in the current trial *n* when the same conflict type was repeated. When the conflict type changed between two consecutive trials, however, no such Gratton effect was observed. Thus, the mechanism responsible for local behavioral adaptation seems to be conflict-type specific; that is, experiencing a Simon conflict in one trial does not affect conflict processing in a subsequent spatial Stroop trial. Second, the manipulation of congruency proportion in the Simon task yielded similar LWPC effects in both tasks. That is, the proportion of congruent trials in the Simon task affected the size of the congruency effect not only in the Simon task itself but also in the spatial Stroop task. This dissociation between local and global effects implies that distinct mechanisms underlie these two phenomena. Specifically, trial-by-trial modulations of the congruency effect could be the sign of a task-specific adaptation mechanism. By contrast, the global behavioral adaptation underlying the LWPC effect points to a task-unspecific mechanism that regulates processing in conflict tasks irrespective of the specific conflict type.

Wühr et al., (2015) observed a similar dissociation between local and global behavioral adaptation effects when Simon and color-Stroop trials were randomly intermixed in a color-discrimination task. Again, sequential adaptation effects were only observed when two trials of the same conflict type were repeated, and local adaptation therefore seemed to be task-specific. In contrast, the LWPC effect caused by a proportion-congruent manipulation for one task type (the inducer task) transferred to the other task type (the diagnostic task), again suggesting that a common mechanism was applied across different conflict tasks. Interestingly, transfer of both local and global adaptation effects across tasks was observed when horizontal and vertical Simon trials were intermixed, with color being the relevant dimension for both tasks. In contrast, neither local nor global adaptation effects transferred across tasks when horizontal and vertical Simon trials were intermixed but the relevant dimensions were different for the two tasks (i.e., color and form). Therefore, the authors suggested that the transfer of LWPC effects across tasks is restricted to cases in which both tasks require responses to the same relevant dimension. On a theoretical level, this seems most consistent with the view that the LWPC effect results from a higher-level mechanism that enacts cognitive control based on enhanced processing of the task-relevant dimension rather than suppression of the task-irrelevant dimension (Wühr et al., 2015, but see Schmidt 2019, for an alternative explanation based on temporal contingencies).

Whereas the studies cited above focused on Simon and Stroop conflicts, the aim of the present study was to examine the effects of global and local behavioral adaptation within and across the Eriksen and Simon tasks. Specifically, in each trial of two experiments, participants responded to the identity of a letter (H or S). Randomly varying from trial to trial, either a Simon or an Eriksen display was presented. In the Simon task, the letter could appear on the left or right side of fixation, whereas in the Eriksen task, the target letter always appeared at fixation and was flanked by two task-irrelevant flanker letters on each side.

In our first experiment, we investigated whether the Simon and Eriksen tasks would be equally affected by a manipulation of congruency proportion that is applied to both tasks. For example, one might assume that the irrelevant spatial information in the Simon task might be less vulnerable to cognitive control processes (i.e., it might be processed more automatically and thus be harder to suppress) than the letter identity information conveyed by the flankers in the Eriksen task. Accordingly, one might expect a smaller effect of the manipulation of congruency proportion in the Simon task than in the Eriksen task. Similarly, sequential adaptation, as is evident in the Gratton effect, might be less pronounced in the Simon task than in the Eriksen task.

In our second experiment, we assessed potential transfer of global and local adaptation across these two tasks. That is, we investigated whether a similar dissociation between LWPC and Gratton effects as reported by Funes et al., (2010) and Wühr et al., (2015) would be found when trials of the Simon and Eriksen tasks are intermixed. Hence, in this experiment, congruency proportion was manipulated for only one of the two tasks (i.e., the inducer task), in order to assess whether the effect of this manipulation would spill over to the other task (i.e., the diagnostic task). Likewise, we assessed whether the magnitude of the Gratton effect would depend on the repetition or change of conflict type from one trial to the next. Since in our setup the target stimulus was identical for both tasks (target letters) while the conflicting information was different (location vs. letter identity), based on the results of Wühr et al., (2015) one might expect transfer of global but not local behavioral adaptation effects. Finding the same pattern of dissociation between LWPC and Gratton effects for intermixed Simon and Eriksen trials as the one previously observed for intermixed Simon and Stroop trials would further support the idea that local and global behavioral adaptation are based on different underlying mechanisms. Moreover, this would also suggest that the same form of global behavioral adaptation can be implemented across all three different conflict types, based on a common mechanism, which presumably enacts cognitive control through enhanced processing of the task-relevant information.

## Experiment 1

Both the Eriksen and Simon tasks were performed in each block of this experiment. Specifically, half of all trials within each block were assigned to the Eriksen task and the other half to the Simon task. The order of trials within each block was randomized, such that each task occurred with a probability of 50 % on each trial. The congruency proportion of all trials varied across blocks, that is, a single block contained either 75 % or 25 % congruent trials, equally for the Eriksen and Simon tasks. If the same mechanism regulates behavioral adaptation in both tasks, irrespective of conflict type, the manipulation of congruency proportion should exert similar LWPC effects on both tasks. If, however, LWPC effects are based on cognitive control and, as speculated above, the task-irrelevant spatial information in the Simon task is less vulnerable to such control processes than the symbolic flanker information in the Eriksen task, the Simon task should be less affected by congruency proportion.

In addition, the experimental setup allowed us to investigate whether local behavioral adaptation (i.e., as indicated by the Gratton effect) exerts similar effects in both tasks, and whether these effects are task-specific or whether they transfer across the two tasks.

### Method

#### Participants

In traditional Eriksen tasks, 6 to 28 participants are usually tested (Servant and Logan, 2019). To ensure high statistical power, we tested 50 participants. Specifically, if one proceeds from the assumption of a medium effect size, *d* = 0.5, this sample size implies a statistical power of 80 % for a paired *t* test with *α* = 0.05 (two-sided test). Two participants had to be excluded due to slow responses (i.e., mean RT greater than 1350 ms, with all other participant means less than 750 ms) and/or especially high error rates (i.e., percentage of correct responses less than 73 %, with all other participant PCs greater than 87 %). The mean age of the remaining 32 female and 16 male participants was 25.23 (*SD* = 5.03). All participants were recruited from the University of Tübingen and received either course credit or 8 €/h in exchange for their participation. All provided informed written consent prior to the experiment.

#### Stimuli and apparatus

The participants were seated in a dimmed and sound-attenuated booth in front of a Fujitsu Lifebook with a viewing distance of approximately 50 cm. All stimuli were generated and presented using PsychoPy 3 (Peirce et al., 2019). A white cross (5 mm × 5 mm) served as fixation and the letters H and S (Arial font, 7 mm high and 6 mm wide) served as the target and flanker stimuli. In addition, the word “Fehler!”(“error!”) served as feedback in case of an erroneous response. All stimuli were displayed in white on a mid-grey background. Responses were made with the left and right index fingers on a German QWERTZ keyboard using the left and right STRG (CTRL) keys. The target letter H was assigned to the left key and the target S to the right key.

#### Procedure

Each trial started with a blank screen for 500 ms, followed by the presentation of the fixation cross at the screen center for another 500 ms. Then, either an Eriksen or a Simon task display appeared and remained on screen until the participant responded. Specifically, in the Eriksen task the target letter appeared at the screen center and it was flanked by four distractor letters, that is, two on each side (left and right). The center-to-center distance between adjacent letters was 1 cm. For the Simon task, a single target letter appeared either 2 cm to the left or to the right of the screen center (that is, at the position occupied by the left- or rightmost distractor in the Eriksen task). Participants were instructed to respond to the identity of the central letter in case of a five-letter (Eriksen) display and to the identity of the single letter in case of a single-letter (Simon) display.

In the Eriksen task, target and distractor identity were either the same (congruent condition, e.g., ‘HHHHH’) or different (incongruent condition, e.g., ‘HHSHH’). In the Simon task, the target either appeared on the same side as the correct response (congruent condition, e.g., ‘H’ presented on the left side) or on the opposite side from the correct response (incongruent condition, e.g., ‘H’ presented on the right side). Following a correct response, the next trial started after 800 ms, during which the screen remained empty. Following an incorrect response, the error message was presented during this 800 ms period. Participants were instructed to respond as quickly as possible and to avoid response errors.

The main session comprised ten blocks of 64 trials each (all presented in random order), that is, 32 trials for the Eriksen task and 32 trials for the Simon task. There were two types of blocks differing in congruency proportion: In ‘mostly congruent’ blocks, the proportion of congruent trials in each task was 75 %, whereas this proportion was 25 % in ‘mostly incongruent’ blocks. Half of the participants performed all of the five ‘mostly congruent’ blocks first and then proceeded to the five ‘mostly incongruent’ blocks. The remaining half of participants received these block types in the reverse order. The first block of each congruency proportion was considered practice and thus excluded from all data analyses. Participants initiated each block when they felt ready to proceed. The design of this experiment was thus *Congruency* (congruent vs. incongruent) × *Current Task* (Flanker vs. Simon) × *Congruency Proportion* (mostly congruent vs. mostly incongruent).

### Results

Trials with incorrect responses (3.8 %), RTs less than 200 ms (0.05 %), or RTs greater than 1500 ms (0.24 %) were excluded from the analyses of RT. To facilitate data presentation, in the following we do not report RT and PC directly but treat the effects of congruency on these measures as dependent variable. Figures and Tables of mean RT and PC data for all experiments, as well as the results of the corresponding ANOVAS on these measures, are available in the Appendix.

In order to investigate the effects of congruency proportion (i.e., LWPC effects) on the congruency effects in the Eriksen and Simon tasks, congruency effects (for RT: *RT*_*incongruent*_ − *RT*_*congruent*_; for PC: *PC*_*congruent*_ − *PC*_*incongruent*_) were calculated for each participant and combination of current task and congruency proportion. The resulting values were then submitted to separate repeated- measures ANOVAs with the factors current task and congruency proportion.

In addition, to investigate sequential modulations of the congruency effect (i.e., the Gratton effect) within and across tasks, data were collapsed across congruency proportions, and split according to task and congruency in the current and the immediately preceding trial. Specifically, trials were regrouped according to whether the current task repeated or switched from the preceding trial and whether the current and the preceding trial was congruent or incongruent. Again, congruency effects for RT and PC were calculated for each participant and combination of current task, previous task, and previous congruency, and submitted to within-subjects ANOVAs with the respective factors.

### List-wide proportion-congruency effects

**Fig. 1:**
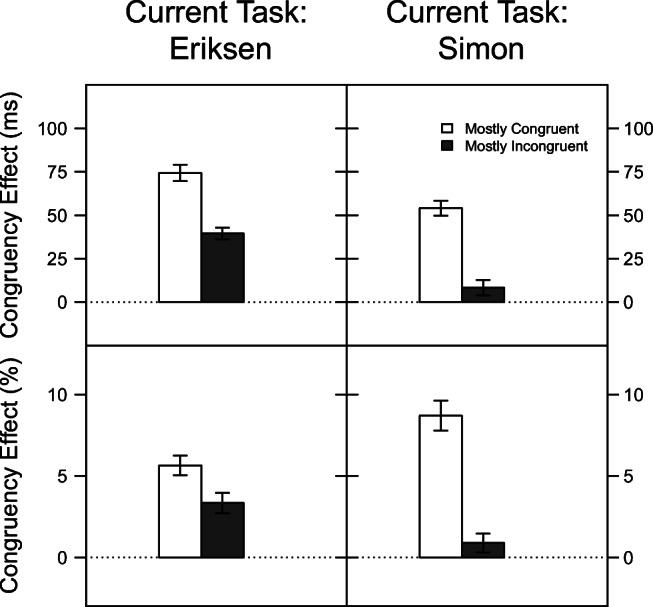
Mean congruency effects in Experiment 1 for reaction time (*top row*) and percentage of correct responses (*bottom row*) as a function of current task and congruency proportion. The LWPC effect corresponds to the difference between adjacent *filled* and *unfilled bars*. The *error bars* reflect ± 1 within-subjects standard error of the mean, computed using the method of Morey (2008)

Figure 1 displays the mean congruency effects on RT (top row) and PC (bottom row) as a function of current task and congruency proportion.

#### Reaction time

A significant main effect of current task revealed a larger congruency effect for the Eriksen task (57 ms) than for the Simon task (31 ms), *F*(1,47) = 35.38, *p* < .001, *η*^2^ = .43. Importantly, the overall congruency effect was larger in mostly congruent blocks (64 ms) than in mostly incongruent blocks (24 ms), *F*(1,47) = 72.38, *p* < .001, *η*^2^ = .61. That is, a typical LWPC effect was observed. This effect was not significantly modulated by current task, *F*(1,47) = 2.44, *p* = .125, *η*^2^ = .05 (cf. Fig. 1, upper rows). In fact, contrary to our hypothesis, numerically, congruency proportion influenced the congruency effect slightly more in the Simon task than in the Eriksen task.

#### Percentage of correct responses

The congruency effect on PC was similar for the Eriksen task (4.49 %) and for the Simon task (4.80 %), *F*(1,47) = 0.20, *p* = .655, *η*^2^ < .01. Mirroring the RT results, a larger congruency effect was observed in blocks with mostly congruent (7.17 %) than with mostly incongruent (2.12 %) trials, *F*(1,47) = 52.32, *p* < .001, *η*^2^ = .53. Interestingly, this LWPC effect was significantly modulated by current task, *F*(1,47) = 14.76, *p* < .001, *η*^2^ = .24. As is evident in the lower panels of Fig. 1, the effect of congruency proportion on PC was much larger in the Simon task than in the Eriksen task.

### Sequential modulation of the congruency effect

**Fig. 2:**
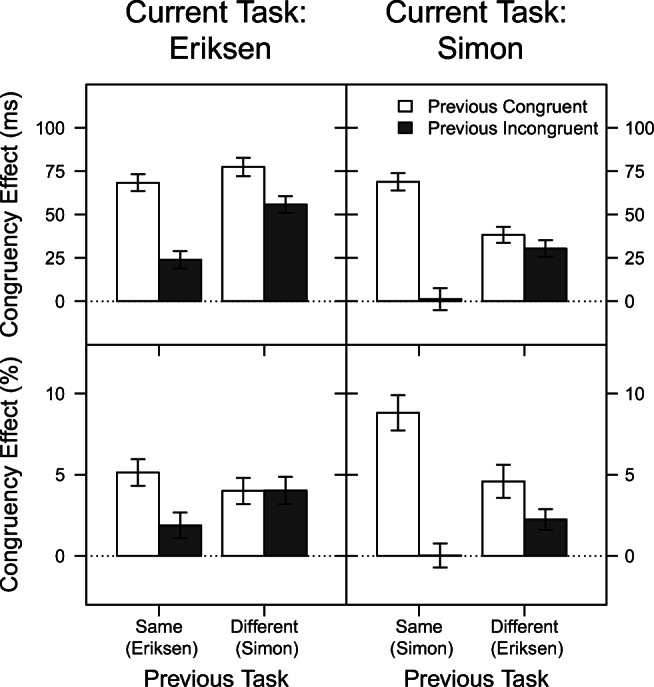
Mean congruency effect in Experiment 1 for reaction time (*top row*) and percentage of correct responses (*bottom row*), as a function of current task, previous task, and previous congruency. For each combination of current task and previous task, the Gratton effect is the difference between the adjacent *filled* and *unfilled bars*. The *error bars* reflect ± 1 within-subjects standard error of the mean, computed using the method of Morey (2008)

Figure 2 displays the mean congruency effects on RT (top row) and PC (bottom row) as a function of current task, previous task, and previous congruency.

#### Reaction Time

As was seen in the top row of Fig. 1 and can be seen again in the top row of Fig. 2, the congruency effect was on average larger for the Eriksen task (56 ms) than for the Simon task (35 ms), leading to a significant effect of current task again in this analysis, *F*(1,47) = 33.44, *p* < .001, *η*^2^ = .42. As expected, the congruency effect in a given trial was larger when the previous trial was congruent (63 ms) than when it was incongruent (28 ms), *F*(1,47) = 58.76, *p* < .001, *η*^2^ = .56. That is, a typical Gratton effect was observed. This Gratton effect did not differ between the two task types (Previous Congruency × Current Task: *F*(1,47) = 0.49, *p* = .488, *η*^2^ = .01), but importantly, it did depend on whether the previous task was the same or different (Previous Congruency × Previous Task: *F*(1,47) = 41.32, *p* < .001, *η*^2^ = .47). Specifically, for task repetitions, the congruency effect reduced from 69 ms to 13 ms when the previous trial was congruent vs. incongruent, respectively (i.e., a pronounced Gratton effect), whereas for task switches, the congruency effect reduced only slightly from 58 ms to 43 ms depending on the previous trial’s congruency.

Also, averaging across previous congruent and incongruent trials, the congruency effect was slightly smaller when the current task repeated (41 ms) rather than changed from the previous trial (51 ms), *F*(1,47) = 9.94, *p* < .003, *η*^2^ = .17. This effect, however, was further modulated by the current task type (Previous Task × Current Task: *F*(1,47) = 9.84, *p* = .003, *η*^2^ = .17). Specifically, it can be attributed exclusively to the Eriksen task, in which the congruency effect on average amounted to 46 ms vs. 67 ms when the task repeated vs. changed from the previous trial, whereas in the Simon task, these values were virtually identical (35 vs. 34 ms for task repetition vs. task change).

Finally, the three-way interaction was also significant (Previous Task × Previous Congruency × Current Task: *F*(1,47) = 6.55, *p* = .014, *η*^2^ = .12). Basically, as can be seen in Fig. 2, the Gratton effect was especially strong for Simon trials preceded by Simon trials, and almost completely abolished for Simon trials preceded by Eriksen trials. For the Eriksen task, a similar but less pronounced pattern emerged. Follow-up ANOVAs conducted on the two tasks separately confirmed interactions of previous congruency and previous task for both the Simon task, *F*(1,47) = 36.35, *p* < .001, *η*^2^ = .44, and the Eriksen task, *F*(1,47) = 5.80, *p* = .020, *η*^2^ = .11. Therefore, for both tasks the Gratton effect reduced when the task changed rather than repeated from the previous trial. However, as indicated by post hoc *t* tests, the Gratton effect in the Eriksen task was still significant after a previous Simon trial, *t*(47) = 2.83,*p* = .007, whereas there was no indication of such an effect in the Simon task after a preceding Eriksen trial, *t*(47) = 1.29,*p* = .202.

#### Percentage of Correct Responses

The congruency effect on PC is depicted in the bottom row of Fig. 2. Again, the congruency effect was on average larger when the previous trial was congruent (5.63 %) than when it was incongruent (2.05 %), *F*(1,47) = 35.54, *p* < .001, *η*^2^ = .43. This Gratton effect depended on the task sequence, *F*(1,47) = 11.54, *p* = .001, *η*^2^ = .20. Specifically, for task repetitions, the congruency effect reduced from 6.97 % to 0.95 % when the previous trial was congruent vs. incongruent, respectively (i.e., a pronounced Gratton effect), whereas for task switches, the congruency effect reduced again only slightly (from 4.29 % to 3.14 %). In addition, an interaction of previous congruency and current task indicated that the Gratton effect was more pronounced in the Simon task (reduction from 6.70 % for previous congruent trials to 1.14 % for previous incongruent trials) than in the Eriksen task (reduction from 4.57 % to 2.96 %), *F*(1,47) = 11.77, *p* = .001, *η*^2^ = .20. None of the other main effects or interactions were significant (all *p*’s > .15).

## Discussion

This experiment was conducted to assess global (i.e., LWPC effects) and local (i.e., Gratton effects) behavioral adaptation within and across the Eriksen and Simon tasks. The main findings can be summarized as follows. First, the proportion of congruent trials affected both tasks, such that the congruency effects in these tasks were substantially reduced in blocks with mostly incongruent vs. mostly congruent trials. This is in line with a number of studies that have demonstrated LWPC effects in Eriksen (e.g., Aben et al., 2017, Corballis and Gratton, 2003, Lehle & Hübner, 2008) and Simon (e.g., Hommel, 1994, Stürmer et al., 2002) tasks, and it extends these findings to a situation in which these two conflict types are randomly (and thus unpredictably) intermixed.

Second, contrary to our hypothesis, this effect was slightly more pronounced in the Simon than in the Eriksen task (significantly so with respect to the proportion of correct responses). This indicates that behavioral adaptation is at least as effective for the task-irrelevant spatial information in the Simon task as for the symbolic information in the Eriksen task. For example, from a cognitive-control perspective, one might say that irrelevant spatial information can be at least as effectively suppressed as irrelevant symbolic information in the case of frequently experienced conflict. In fact, suppressing the symbolic flanker information in the Eriksen task even seems to be slightly more difficult. A (speculative) explanation for this finding could be that intermixing of Simon and Eriksen trials might have broadened the attentional focus (Eriksen & St. James, 1986), since in 50 % of trials (i.e., the Simon trials), the outermost flanker positions contained the target stimulus (see also Aben et al., 2019, for a demonstration of sustained effects of attentional focus in a conflict task). Consequently, relative to a pure Eriksen paradigm, processing of the flanker letters in Eriksen trials of the present design might have been enhanced and thus they might have impacted on processing rather strongly, even in blocks with mostly incongruent trials. Given that the difference between the LWPC effects in the Simon and Eriksen task, however, is rather small and significant only for response accuracy, there is no strong evidence for clear qualitative differences in the mechanisms responsible for list-wide behavioral adaptation in the two tasks.

Third, we also observed sequential modulations of the congruency effect in both tasks. Specifically, pronounced Gratton effects were observed for Simon and Eriksen trials when these were preceded by the same task type. Again, this is consistent with previous studies investigating local behavioral adaptation effects in the Eriksen (e.g., Gratton et al., 1992) and the Simon task (e.g., Hommel et al., 2004, Stürmer et al., 2002), and extends these findings to a situation where the two tasks are intermixed. Similar to the LWPC effects reported above, the Gratton effect seems more pronounced in the Simon than in the Eriksen task. In fact, the congruency effect was completely absent in Simon trials following incongruent Simon trials, indicating especially effective behavioral adaptation following a recent similar conflict trial. Even though less pronounced, the same was true for the Eriksen task—congruency effects were strongly reduced, though not completely abolished, following incongruent trials of the same task type.

Fourth, the Gratton effect was strongly reduced when the task changed between the preceding and the current trial, suggesting that the mechanism underlying this local behavioral adaptation is largely task-specific. Similar results have been, for example, reported by Wendt et al., (2006), who kept congruency proportion at 50 % throughout the experiment while alternating between Simon and Eriksen trials. Yet, a slight asymmetry is again evident regarding the two task types: While the Gratton effect on RT was reduced in the Eriksen task after task switches compared to task repetitions, it was not completely absent, as it appeared to be in the Simon task. That is, at least to a certain extent, experiencing a Simon conflict may trigger behavioral adaptation that may help reduce a subsequent Eriksen conflict. As a secondary finding, the observed effects of congruency in the Eriksen task were especially large after preceding Simon compared to Eriksen trials, irrespective of whether these preceding trials were incongruent or congruent. Again, this fits quite well with the tentative explanation of an enlarged attentional focus following Simon trials as outlined above (i.e., enhanced flanker processing after processing a target stimulus at a flanker position). Note, however, that this would constitute a more general attentional phenomenon rather than a specific mechanism dedicated to selective adjustment of cognitive control after a conflict is experienced.

In sum, the mechanism responsible for local behavioral adaptation in this experiment appeared largely (even though not completely) task-specific, when considering sequential modulations across the Eriksen and Simon task. However, global behavioral adaptation afforded by the manipulation of congruency proportion in both tasks appeared quite similar. Therefore, it is conceivable that the same mechanism underlying global adaptation effects, may it be conflict-based or contingency based, operates on the two tasks. Yet, a stricter test of the specificity vs. generality of global behavioral adaptation would require a design that enables assessing potential transfer of the LWPC effect across the two tasks. To meet this requirement, in Experiment 2 we manipulated congruency proportion for only one of the two tasks, and tested whether and to what extent this manipulation also affected the congruency effect in the other task.

## Experiment 2

This experiment was similar to Experiment 1, except that for each participant the frequency of congruent trials was only manipulated for one task (i.e., only for the Simon task but not for the Eriksen task, or vice versa). Following Wühr et al., (2015), we will refer to the task in which congruency proportion was manipulated as the “inducer” task and the task in which congruency proportion was always 50 % as the “diagnostic” task (see also Braem et al., 2019). As in Experiment 1, each block was comprised of trials from both tasks. There were two groups of participants: In the Eriksen inducer group, the proportion of congruent trials among all Eriksen trials (inducer task) within a block was either 16.7 % or 83.3 %, whereas the proportion of congruent trials among all Simon trials (diagnostic task) was always 50 %. In the Simon inducer group, the proportion of congruent trials among all Simon trials (inducer task) was either 16.7 % or 83.3 % whereas the proportion of congruent trials among all Eriksen trials (diagnostic task) always 50 %. The major question was whether the manipulation of congruency proportion in the inducer task would modulate the congruency effect in both tasks, as one might expect if the two tasks were supervised by a common global adaptation mechanism based on conflict regulation. However, if task- or stimulus-specific mechanisms evoke the conflict adaption reflected in the LWPC effect, then there should be no spillover of the LWPC effect from the inducer task to the diagnostic task. Likewise, we again examined whether sequential behavioral adaptation (i.e., as indicated by the Gratton effect) is task-specific or whether it transfers across the two tasks. Based on the results of Experiment 1, we would again expect little or no transfer of this effect across tasks.

### Method

#### Participants

In order to achieve high statistical power, 48 participants were tested for each of the two groups. Four participants of the original sample were replaced due to especially slow responses and / or high error rates well outside the range of the remaining participants. The mean age of the final sample, consisting of 80 female and 16 male participants, was 21.7 (*SD* = 4.5) years.

#### Stimuli and Apparatus

The stimuli and apparatus were identical to those employed in Experiment 1.

#### Procedure

The procedure was identical to that of Experiment 1 with the following exceptions. For half of the participants, the Eriksen task served as inducer task and for the other half, the Simon task served as inducer. In contrast to Experiment 1, the proportion of congruent and incongruent trials differed between the inducer and diagnostic task (see Table 1). Across tasks, the overall percentages of congruent and incongruent trials were identical to Experiment 1, that is, 75% in “mostly congruent” blocks and 25% in “mostly incongruent” blocks. Each participant completed seven successive blocks for each Congruency Proportion, that is, 14 blocks in total. As in Experiment 1, the order of these two block types was counterbalanced across participants, and the first block of each block type was considered practice and thus was not included in the data analysis. Experiment 2 thus had a mixed design with the within-subjects factors of *Congruency* (congruent vs. incongruent), × *Current Task* (Eriksen vs. Simon) × *Congruency Proportion of the Inducer Task* (mostly congruent vs. mostly incongruent) and the between-subjects factor of *Inducer Task* (Eriksen vs. Simon).

**Table 1:** Trial frequency and congruency proportion for a single block of each block type in Experiment 2

		Congruency Proportion Block			
		Mostly Congruent		Mostly Incongruent	
Task	Congruency	N^a^	*%*^b^	N^a^	*%*^b^
Inducer					
	Congruent	40	83.3	8	16.7
	Incongruent	8	16.7	40	83.3
Diagnostic					
	Congruent	8	50.0	8	50.0
	Incongruent	8	50.0	8	50.0
	Sum	64		64	

^a^ Number of trials per block.

^b^ Percentage of congruent vs. incongruent trials within each task.

### Results

Trials with incorrect responses and trials with RTs less than 200 ms (0.01 %), or RTs greater than 1500 ms (0.13 %) were excluded from the analyses of RT. Congruency effects on RT and PC were computed as in Experiment 1 for each participant and combination of current task, inducer task and congruency proportion. The resulting values were then submitted to separate mixed-design ANOVAs with the within-subject factors current task (Eriksen vs. Simon) and congruency proportion (mostly congruent vs. mostly incongruent) and the between-subjects factor inducer task (Eriksen vs. Simon).

In addition, to investigate sequential modulations of the congruency effect (i.e., the Gratton effect) within and across tasks, data were collapsed across congruency proportions, but split according to inducer task, current task, previous task, and congruency in the immediately preceding trial. Specifically, trials were regrouped according to whether the current task repeated or switched from the preceding trial and whether the preceding trial was congruent or incongruent. Again, congruency effects on RT and PC were calculated for each participant and combination of current task (Eriksen vs. Simon), previous task (same vs. different) and previous congruency (congruent vs. incongruent), and submitted to mixed-design ANOVAs with inducer task (Eriksen vs. Simon) as an additional between-subjects factor.

### List-wide proportion-congruency effects

**Fig. 3:**
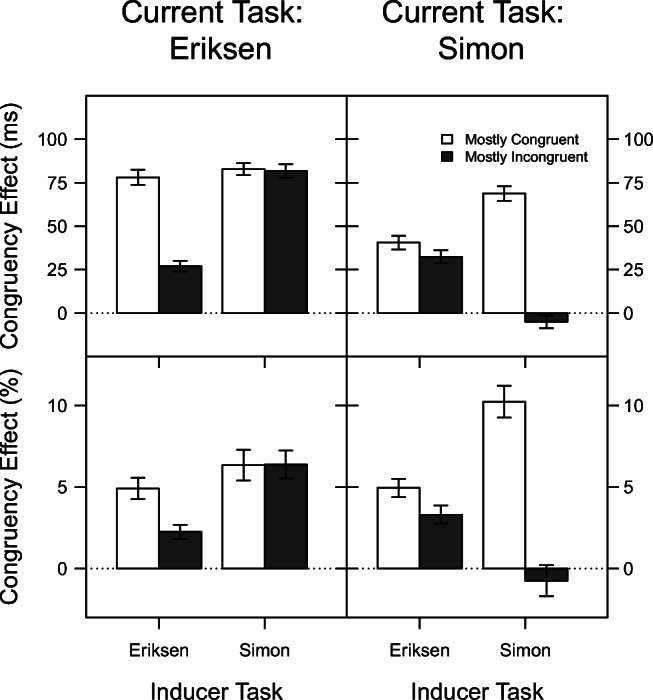
Mean congruency effect in Experiment 2 for reaction time (*upper panels*) and percentage of correct responses (PC, *lower panels*) as a function of current task, congruency proportion, and inducer task. The LWPC effect corresponds to the difference between adjacent *filled* and *unfilled bars*. The *error bars* reflect ± 1 within-subjects standard error of the mean, computed using the method of Morey (2008)

Figure 3 displays the congruency effects on RT (top row) and PC (bottom row) as a function of current task, congruency proportion, and inducer task.

#### Reaction time

Overall, the congruency effect on RT was larger for the Eriksen task (67 ms) than for the Simon task (34 ms), *F*(1,94) = 126.13, *p* < .001, *η*^2^ = .57. Also, it was on average larger when the Simon task served as inducer (57 ms) than when the Eriksen task (45 ms) served as inducer, *F*(1,94) = 12.99, *p* = .001, *η*^2^ = .12. These two factors interacted, *F*(1,94) = 33.82, *p* < .001, *η*^2^ = .26, reflecting that the congruency effect was especially large for Eriksen trials when these served as diagnostic task (82 ms), compared to when they served as inducer task (53 ms), while the congruency effect in Simon trials was only slightly larger when these were diagnostic (37 ms) than when they served as inducer (32 ms).

As expected, the congruency effect was larger in blocks with mostly congruent (68 ms) than in blocks with mostly incongruent (34 ms) trials, *F*(1,94) = 160.08, *p* < .001, *η*^2^ = .63. This LWPC effect was more pronounced for the Simon task than the Eriksen task, *F*(1,94) = 9.41, *p* = .003, *η*^2^ = .09. Specifically, when the proportion of incongruent trials increased, the congruency effect reduced from 80 ms to 54 ms for the Eriksen task and from 55 ms to 14 ms for the Simon task.

The interaction of inducer task and congruency proportion was not significant, that is, the overall LWPC effect was similar irrespective of which task served as inducer, *F*(1,94) = 2.15, *p* = .146, *η*^2^ = .02. Theoretically, most interesting was the threefold interaction of Inducer Task × Current Task × Congruency Proportion, *F*(1,94) = 141.44, *p* < .001, *η*^2^ = .60. As can be seen in Fig. 3, the LWPC effect (i.e., the difference between the congruency effects in mostly congruent and mostly incongruent blocks) was especially pronounced for the Simon (74 ms) and the Eriksen (51 ms) task when these tasks served as inducer, and almost completely abolished when these tasks served as diagnostic (Simon: 8 ms, Eriksen: 1 ms). In essence, this means that the LWPC effect was nearly or completely specific to the task in which congruency proportion was varied, with little or no transfer to the task in which congruency proportion was always 50 %. This latter conclusion was substantiated by an additional post-hoc ANOVA with factor congruency proportion, conducted on the congruency effect in diagnostic trials only. This analysis showed no effect of congruency proportion in diagnostic trials, *F*(1,95) = 1.64, *p* = .204, *η*^2^ = .02.

#### Percentage of correct responses

The congruency effect on PC did not differ significantly between the Simon (4.43 %) and Eriksen (4.97 %) tasks, *F*(1,94) = 0.65, *p* = .422, *η*^2^ = .01. It was, however, slightly larger when the Simon task served as inducer (5.55 %) than when the Eriksen task served as inducer, (3.85 %), *F*(1,94) = 4.25, *p* = .042, *η*^2^ = .04. There was no interaction between these two factors, *F*(1,94) = 2.70, *p* = .104, *η*^2^ = .03. However, congruency proportion and all interactions including this factor modulated the congruency effect (Proportion: *F*(1,94) = 64.26, *p* < .001, *η*^2^ = .41; Current Task × Proportion: *F*(1,94) = 27.82, *p* < .001, *η*^2^ = .23; Inducer Task × Proportion: *F*(1,94) = 12.12, *p* = .001, *η*^2^ = .11; Current Task × Inducer Task × Proportion: *F*(1,94) = 40.26, *p* < .001, *η*^2^ = .30). Summarizing these effects, on average the congruency effect was strongly reduced in blocks with mostly incongruent (6.61 %) versus mostly congruent trials (2.79 %), but this LWPC effect was moderated strongly by current task and inducer task. Specifically, the LWPC effect was especially pronounced for the Simon (10.98 %) and the Eriksen (2.66 %) task when these tasks served as inducer, and almost completely abolished when these tasks served as diagnostic (Simon: 1.65 %, Eriksen: -0.04 %). Similar to the additional analysis above, this was substantiated by an additional post-hoc ANOVA with factor congruency proportion, conducted on the congruency effect in diagnostic trials only. As for the congruency effect on RT, this analysis showed no effect of congruency proportion in diagnostic trials, *F*(1,95) = 1.57, *p* = .214, *η*^2^ = .02.

### Sequential modulation of the congruency effect

#### Reaction time

**Fig. 4:**
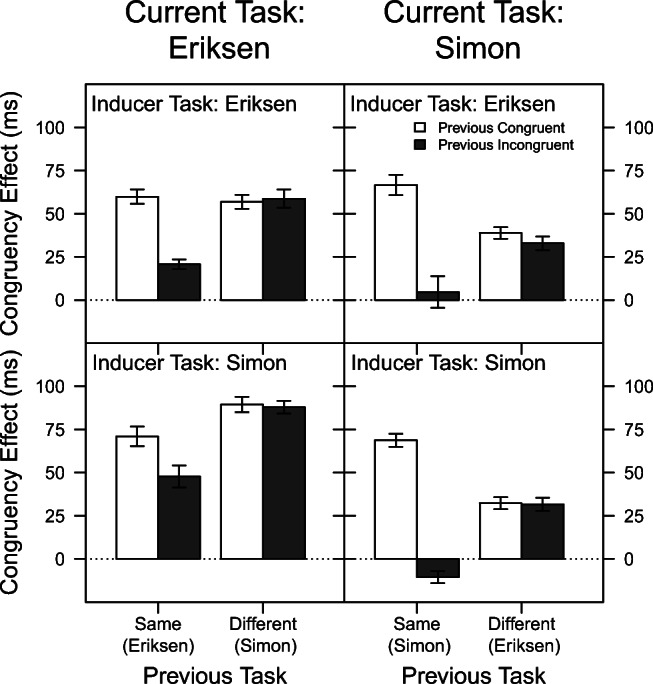
Mean congruency effect in Experiment 2 for reaction time as a function of current task, previous task, previous congruency, and inducer task. The Gratton effect is the difference between the adjacent *filled* and *unfilled bars*. The *error bars* reflect ± 1 within-subjects standard error of the mean, computed using the method of Morey (2008)

Figure 4 displays the mean congruency effect on RT as a function of current task, previous task, previous congruency, and inducer task. As can be seen in this figure, the basic pattern of results resembles the one observed in Experiment 1. Again, the congruency effect was on average larger for the Eriksen task (62 ms) than for the Simon task (33 ms), *F*(1,94) = 78.11, *p* < .001, *η*^2^ = .45. Also, the congruency effect was on average slightly smaller when the task repeated from the previous trial (41 ms) than when it changed (54 ms), *F*(1,47) = 28.71, *p* < .001, *η*^2^ = .23. This effect, however, was again further modulated by the current task type (Previous Task × Current Task), *F*(1,94) = 31.06, *p* < .001, *η*^2^ = .25. As in Experiment 1, it can be attributed exclusively to the Eriksen Task, in which the congruency effect on average amounted to 50 ms vs. 73 ms when the task repeated vs. changed from the previous trial, whereas in the Simon task, these values were virtually identical (32 vs. 34 ms respectively).

As expected, the congruency effect was on average larger when the previous trial was congruent (60 ms) than when it was incongruent (34 ms), *F*(1,94) = 114.68, *p* < .001, *η*^2^ = .55. That is, a typical Gratton effect was again observed. In contrast to Experiment 1, this effect differed between the two task types (Previous Congruency × Current Task: *F*(1,94) = 25.84, *p* < .001, *η*^2^ = .22), that is, it was larger in the Simon task (52 vs. 15 ms for previous congruent vs. previous incongruent) than in the Eriksen task (69 vs. 54 ms). Most importantly, the Gratton effect again depended also on whether the previous task was the same or different (Previous Congruency × Previous Task: *F*(1,94) = 95.71, *p* < .001, *η*^2^ = .50). Specifically, for task repetitions, the congruency effect reduced from 67 ms to 16 ms when the previous trial was congruent vs. incongruent (i.e., a pronounced Gratton effect), whereas for task switches, the congruency effect was virtually identical whether the previous trial was congruent (54 ms) or incongruent (53 ms).

Also similar to Experiment 1, the three-way interaction of Previous Task × Previous Congruency × Current Task was significant, *F*(1,94) = 17.81, *p* < .001, *η*^2^ = .16. Again, the Gratton effect (i.e., the difference between the congruency effects in previous congruent and previous incongruent trials) was especially strong for Simon trials preceded by Simon trials (71 ms), and almost completely abolished for Simon trials preceded by Eriksen trials (3 ms). For the Eriksen task, a similar but less pronounced pattern emerged (31 vs. 0 ms). The apparent lack of Gratton effects following a change of the task type was substantiated by an additional mixed-design ANOVA with factors current task, previous congruency and inducer task, conducted only on trials which were preceded by a different task type. This analysis showed that neither the main effect of previous congruency nor any interaction involving this factor (all *ps* > .35) reached significance. Therefore, no Gratton effects could be observed following a task switch.

Finally, the congruency effect also differed depending on which task served as the inducer task, *F*(1,94) = 8.13, *p* = .005, *η*^2^ = .08. Specifically, it was overall larger when the Simon task served as inducer (52 ms) than when the Eriksen task served as inducer (42 ms). This factor was also involved in two significant interactions (Current Task × Inducer Task: *F*(1,94) = 22.04, *p* < .001, *η*^2^ = .19; Current Task × Previous Task × Previous Congruency × Inducer Task: *F*(1,94) = 6.03, *p* = .016, *η*^2^ = .06). As can be seen in Fig. 4, these interactions reflect that the Gratton effect, and thus also its reduction after a task switch versus a task repetition, seemed less pronounced especially when the Eriksen task served as diagnostic task. No other interactions involving the factor inducer task reached significance (all *p*’s > .12).

#### Percentage of correct responses

**Fig. 5:**
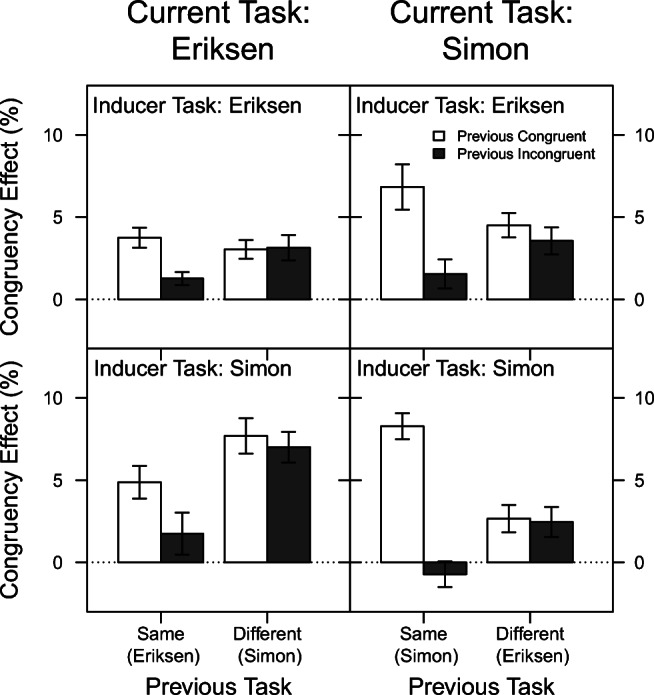
Mean congruency effect in Experiment 2 for percentage of correct responses as a function of current task, previous task, previous congruency, and inducer task. The Gratton effect is the difference between the adjacent *filled* and *unfilled bars*. The *error bars* reflect ± 1 within-subjects standard error of the mean, computed using the method of Morey (2008)

Figure 5 displays the mean congruency effect on PC as a function of current task, previous task, previous congruency, and inducer task. As can be seen in this figure, the pattern of results resembles the one observed for RT and also the results of Experiment 1. On average, the congruency effect neither differed between the two tasks, *F*(1,94) = 0.43, *p* = .512, *η*^2^ < .01, nor depending on which task served as inducer task, *F*(1,94) = 1.19, *p* = .277, *η*^2^ = .01. However, the congruency effect on PC tended to be smaller when the task repeated from the previous trial (3.45 %) than when it changed (4.26 %), *F*(1,94) = 3.40, *p* = .068, *η*^2^ = .03. This effect was again further modulated by the current task type (Previous Task × Current Task: *F*(1,94) = 9.61, *p* = .003, *η*^2^ = .09). As in Experiment 1, it can be attributed exclusively to the Eriksen task, in which the congruency effect on average amounted to 2.91 % vs. 5.22 % when the task repeated vs. changed from the previous trial, whereas in the Simon task, these values were similar (3.98 vs. 3.30 %, respectively).

As expected, the congruency effect in a given trial was larger when the previous trial was congruent (5.20 %) than when it was incongruent (2.50 %), *F*(1,47) = 45.21, *p* < .001, *η*^2^ = .32. That is, a typical Gratton effect was also observed for the analysis of PC. As in the above analysis on the congruency effect on RT, this effect differed between the two task types (Previous Congruency × Current Task: *F*(1,94) = 10.79, *p* < .001, *η*^2^ = .10), that is, it was larger in the Simon task (5.57 vs. 1.71 % for previous congruent vs. previous incongruent) than in the Eriksen task (4.84 vs. 3.29 %).

Importantly, the Gratton effect again also depended on whether the previous task was the same or different (Previous Congruency × Previous Task: *F*(1,94) = 35.08, *p* < .001, *η*^2^ = .27). Specifically, for task repetitions, the congruency effect reduced from 5.93 % to 0.96 % when the previous trial was congruent vs. incongruent, respectively (i.e., a pronounced Gratton effect), whereas for task switches, the congruency effect was similar whether the previous trial was congruent (4.48 ms) or incongruent (4.04 ms).

Also similar to Experiment 1, the three-way interaction of Previous Task × Previous Congruency × Current Task was significant, *F*(1,94) = 8.40, *p* = .005, *η*^2^ = .08. Again, the Gratton effect (i.e., the difference between the congruency effects in previous congruent and previous incongruent trials) was especially strong for Simon trials preceded by Simon trials (7.15 %), and almost completely abolished for Simon trials preceded by Eriksen trials (0.58 %). For the Eriksen task, a similar but less pronounced pattern emerged (2.80 vs. 0.29 ms). Again, an additional mixed-design ANOVA with factors current task, previous congruency and inducer task was conducted only on trials which were preceded by a different task type. This analysis again showed that neither the main effect of previous congruency nor any interaction involving this factor (all *ps* > .30) reached significance. Therefore, also for PC no Gratton effect could be observed following a task switch.

Finally, the factor inducer task did not influence the congruency effect on PC, *F*(1,94) = 1.19, *p* = .277, *η*^2^ = .01. This factor was, however, involved in two significant interactions (Current Task × Inducer Task: *F*(1,94) = 7.37, *p* = .008, *η*^2^ = .07, and Current Task × Previous Task × Inducer Task: *F*(1,94) = 5.54, *p* = .022, *η*^2^ = .05). As can be seen in Fig. 5, these interactions reflect basically that the congruency effect was especially large for a given task when this task was diagnostic rather than inducer, and especially so after a task switch rather than a task repetition. No other interactions involving inducer task reached significance (all *ps* > .10), indicating that the Gratton effect was not much affected by this manipulation.

## Discussion

This experiment was designed to assess global as well as local behavioral adaption effects within and across the Eriksen and the Simon task. The theoretically most important results can be summarized as follows: First, regarding only cases in which the respective task served as inducer, the proportion of congruent trials affected the magnitude of the congruency effect. As in Experiment 1, this LWPC effect was evident for both tasks, and more pronounced in the Simon task than in the Eriksen task. From a cognitive-control perspective, this would suggest that the task-irrelevant information impacts relatively less on the processing of task-relevant information when it conflicts frequently with the relevant information and/or impacts relatively more on task-relevant information when it is frequently consistent with it. Alternatively, differential stimulus-response contingencies, as outlined in the Introduction, might have produced this pattern of results without any dedicated conflict adaptation. In any case, the underlying mechanism seems even more effective in case of the location-based task-irrelevant information of the Simon task than in case of the identity-based task-irrelevant information of the Eriksen task.

Second, there was no evidence for such an LWPC effect in either task when it served as the diagnostic task, that is, when the manipulation of congruency proportion was confined to the other task. Therefore, the behavioral adaptation following the frequent presentation of either congruent or incongruent trials seems to be clearly task-specific, such that frequently presenting spatially incongruent (congruent) information in the Simon task modulates the effects of the spatially-based conflict in the Simon task, but not of the identity-based conflict in the Eriksen task (and vice versa).

Third, interestingly, there again appears to be a certain asymmetry of the LWPC effect between the two tasks. Specifically, congruency effects in the diagnostic Simon trials are approximately halfway between those of the mostly congruent and the mostly incongruent inducer Simon trials. That is, after frequent presentation of incongruent trials, the impact of the irrelevant information reduced, and in response the frequent experience of congruency, the impact of the irrelevant information increased, both to a similar extent. In contrast, in diagnostic Eriksen trials, congruency effects were especially pronounced and of similar magnitude as the ones observed in mostly congruent inducer Eriksen trials. Accordingly, one might conclude that frequent experience of conflict in the Eriksen task enables the suppression of the conflicting flanker information, whereas frequent experience of congruency does not evoke a corresponding increase in the impact of the flanker information (i.e., no processing benefit). On the one hand, this could be taken as another qualitative difference in behavioral adaptation underlying the LWPC effect in the Eriksen and Simon tasks. On the other hand, as suggested in the Discussion of Experiment 1, this difference could also be interpreted by taking the intermixing of the two tasks into account. Again, the frequent need to respond to stimuli at the outermost flanker positions (i.e., in Simon trials) might have broadened the attentional focus in the sense of a “zoom lens” model of attention (Eriksen and St. James, 1986). This could lead to increased flanker processing and correspondingly especially pronounced congruency effects in the Eriksen task. In this case, this specific result pattern could be attributed to attentional factors rather than to specific differences in the mechanisms responsible for cognitive control. This could be further investigated by comparing the relative magnitudes of congruency effects for high, low, and balanced (i.e., 50 %) congruency proportions, when the two tasks are presented in separate blocks rather than intermixed. In this case, potential changes of the attentional focus due to intermixing of the different task types would be prevented, and the effects of cognitive control regarding flanker suppression and flanker enhancement could be reassessed.

Fourth, the basic results regarding sequential adaptation effects remarkably resembled those obtained in Experiment 1. Again, Gratton effects were observed for Simon and Eriksen trials when these were preceded by the same task type, and these effects were more pronounced in the Simon than in the Eriksen task. Moreover, Gratton effects were strongly reduced when the conflict type changed between the preceding and the current trial. In this experiment, the Gratton effect was even completely absent after task switches from the previous trial, and therefore it can be concluded that the mechanism underlying this local adaptation to congruency sequence is task-specific. In addition, the same asymmetry as noted above was again observed between tasks: the observed effects of congruency in the Eriksen task were especially large after preceding Simon compared to Eriksen trials, especially when the Eriksen task was diagnostic (and thus rather infrequently presented). Again, the same explanations may apply to this result, and further light could be shed on it by testing each task in isolation and comparing the results to the task-mixing conditions of the present experiment.

In sum, however, the main research questions of this experiment seem to be answered quite clearly: Local behavioral adaptation (as indexed by the Gratton effect) as well as global behavioral adaptation (as indexed by the LWPC effect) in the Simon and the Eriksen task appear to be completely task-specific, since both effects are clearly evident when regarded within each task, but neither effect transfers across tasks.

## General Discussion

In the present study we investigated the effects of local and global behavioral adaptation in the Simon and Eriksen tasks. In both experiments of this study, we observed typical congruency effects (i.e., longer RT and fewer correct responses) when either the spatial position (Simon task) or the flanking identity information (Eriksen task) was incongruent rather than congruent with the responses afforded by the task-relevant stimulus dimension. These congruency effects were modulated by the sequence of congruent and incongruent trials (i.e., Gratton effects) and by the proportion of congruent trials within a block (i.e., LWPC effects). Specifically, congruency effects were reduced when there was a conflict in the immediately preceding trial, and when conflict trials were especially frequent across a block of trials — indicating that behavioral adaptation effectively took place. Most importantly, however, these modulations could only be observed within each task type but not across task types. Since it has been suggested that the Gratton and LWPC effects may be evoked by two distinct mechanisms which differ regarding their task-specificity (Funes et al., 2010; Wühr et al., 2015), in the following, we will discuss our results with respect to this distinction.

### Local behavioral adaptation

Existing empirical evidence regarding the specificity of local behavioral adaptation is somewhat mixed. In most cases, the Gratton effect seems to be specific to repetitions of the same conflict and task type (for an overview, see Braem, 2014). The task-specific Gratton effects observed here thus fit well within the bulk of existing evidence. Most comparably to the present study, participants in Wendt et al. (2006, Experiment 3) performed Simon and Flanker trials in alternation. In this setup, Gratton effects also occurred only within, but not across the different conflict types.

Interestingly, however, some studies also report Gratton effects that generalize across different conflict types, in cases in which there is substantial overlap in task or response sets. For example, transfer of Gratton effects across conflict types has been observed when the relevant stimulus dimension was shared across conflict types (e.g., target orientation as relevant dimension in Simon and SNARC conflicts, Notebaert & Verguts, 2008, arrow direction as a relevant dimension in Simon and prime-target conflicts, Kunde & Wühr, 2006), or when both tasks require the same response hand and response set (e.g., same response set in Simon and spatial Stroop trials, Lee & Cho, 2013). Even within a single conflict type (e.g., Simon conflict), generalization of the Gratton effect can only be observed when the response set and relevant dimension overlap (Akçay & Hazeltine, 2008; Wühr et al., 2015).

Notably, even though in the present study, as in Wendt et al., (2006), the relevant target dimension (letter identity) and response assignments completely overlapped for the two conflict types, we did not observe generalization of the Gratton effect across the Eriksen and Simon tasks. This underlines the idea that the source of irrelevant information plays an important role in the question of whether behavioral adaptation effects may transfer across different conflict types. Specifically, as already noted by Akçay and Hazeltine (2008): “One critical factor is the nature of the information that is causing the conflict and hence the control processes recruited to deal with it” (p. 973). That is, generalization of Gratton effects across different conflict types may require dimensional overlap not only in task-relevant properties of target and task set, but also in the irrelevant conflicting information. For example, behavioral adaptation may transfer across different conflict types when both are based on spatial conflicting information, but not—as in the present case—when one type of conflict is spatial and the other based on stimulus identity (see also Braem, 2014; Wühr et al., 2015).

### Global behavioral adaptation

Regarding more global behavioral adaptation as indicated by the LWPC effect, a different result may have been expected based on existing literature: As outlined in the Introduction, transfer of LWPC effects across tasks has been demonstrated even under conditions in which local adaptation did not transfer between conflict types. As outlined in the Introduction, transfer of global but not local behavioral adaptation was observed when Simon and spatial Stroop trials (Funes et al., 2010) were randomly intermixed, and when Simon and color-Stroop trials (Wühr et al., 2015) were randomly intermixed. Moreover, in the latter study global transfer was only observed when the task-relevant dimension (e.g., color discrimination) was the same for both tasks. This led the authors to conclude that the adaptation to congruency proportion in the LWPC effect may manifest as heightened attention to the relevant (target) dimension, rather than suppression of the irrelevant information.

On this empirical basis, transfer of the LWPC effect across conflict types might also have been expected in the present study, because both conflict types shared the same target dimension (i.e., letter identity). Furthermore, as in Funes et al., (2010), task set overlap was especially strong since the same target stimuli and target-response assignments were used for both conflict types. Thus, if introducing a high proportion of congruent trials for one conflict type enables heightened attention to and consequently enhanced processing of the target dimension, relative to the irrelevant dimension, this should have become evident in a reduced congruency effect for both conflict types; yet, no such transfer was observed. Even though it is unclear what exactly prevented the LWPC effect from transferring across task types, some possibilities may be discussed in this regard.

First, in contrast to the studies cited above, the present study included an Eriksen task. The conflict in this task differs from the Simon and Stroop-type conflicts combined in previous studies in that the conflict dimension conveyed by the flanker stimuli is identical to the task-relevant dimension of the target stimuli. Consequently, selecting the target dimension (here, letter identity) for enhanced processing in response to frequently encountered conflict might even have the paradoxical effect of also enhancing the processing of the flanker stimuli (a similar argument would apply to a mechanism based on enhancing or suppressing the activations from distractor stimuli as a means of cognitive control). Therefore, this proposed mechanism of cognitive control might—at least for the Eriksen flanker task—prove detrimental to performance and conflict regulation. This difference in the nature of the task-irrelevant information might also explain why behavioral adaptation was slightly more pronounced in the Simon task than the Eriksen task.^1^ However, given that we did observe a decreased congruency effect in the mostly incongruent condition (i.e., a typical LWPC effect) within the flanker task, it is evident that behavioral adaptation successfully took place.

Therefore, one may conclude that this adaptation was presumably based on aspects of the stimulation other than the common target dimension specified in the task sets. A rather striking difference between the two conflict types is the spatial layout of trials, with a single target letter located laterally from fixation in Simon trials and a central target flanked by two letters on each side in Eriksen trials. Thus, target position may have formed the basis for conflict-specific cognitive control. In fact, several studies have demonstrated that within a single conflict type, behavioral adaptation can depend specifically on stimulus context (for an overview, see Bugg and Crump, 2012). For example, Crump et al., (2006) investigated LWPC effects in a Stroop task in which the task-irrelevant information (a color word printed in white) was presented at the screen center and followed by the task-relevant information (a colored patch) appearing either above or below the screen center. Congruency proportion varied depending on the location of the task-relevant information. For example, the color of the patches presented above the screen center was congruent with the color word in most trials, and the color of patches below the screen center was incongruent with the color word in most trials. Thus, target location was indicative of congruency proportion. Crucially, this manipulation affected the magnitude of the congruency effect, such that a larger congruency effect was observed for targets presented at mostly congruent locations than for targets presented at mostly incongruent locations. This modulation, termed the context-specific proportion congruency (CSPC) effect, therefore indicates that even within the same conflict type, target location can be an effective determinant of the degree of behavioral adaptation to conflict proportion. Such CSPC effects have been repeatedly demonstrated, for example, also for the flanker task (e.g., Corballis and Gratton, 2003, Wendt et al., 2008) and for other context-defining features such as color or shape (Crump et al., 2008; Lehle & Hübner, 2008).

Based on these results, one may interpret the present findings as a location-based CSPC effect rather than a task-specific LWPC effect. Therefore, on a theoretical level, transfer of the LWPC effect may not have been prevented because of the different conflict types, but because of the different target locations associated with the two conflict types. Yet, this interpretation does not fit well with the results of Funes et al., (2010) and Wühr et al., (2015), in which target locations also differed systematically between Simon and spatial Stroop trials, but transfer of the LWPC effect was clearly observed.

Further light could be shed on this issue by de-confounding target location and conflict type, for example by presenting the five-letter flanker displays at the same lateral positions as the Simon stimuli. In this case, the single-letter Simon stimuli could be even omitted completely, since each five-letter display would entail both conflict types (Simon and Eriksen conflict). This would further increase dimensional overlap between the two conflict types, and congruency as well as congruency proportion of these conflict types could be varied and analysed orthogonally. In fact, however, such “factorial” designs, as opposed to the “task-switching” design employed in the present study (cf. Braem et al., 2014, for this distinction), have also mostly produced evidence for task-specific behavioral adaptation rather than transfer across tasks (see also Wendt et al., 2006). Another possibility would be to repeat the present study with additional “neutral” Simon trials (i.e., single target stimuli presented at the screen center). If global adaptation effects were due to an enhanced processing of the target dimension at a specific location, rather than suppression of the conflicting information, LWPC effects should transfer from the Eriksen trials to these neutral Simon trials, but not to the laterally presented ones.

A less striking difference between the two conflict types employed in the present study lies in the temporal unfolding of the respective congruency effects. For example, the Simon effect is typically assumed to emerge rather fast and automatically, but decays quickly as RT prolongs (Hommel, 1994; Simon et al., 1976; Vallesi and Umiltà, 2009). This becomes especially evident when the magnitude of the congruency effect is plotted for consecutive bins of the RT distribution (i.e., a delta plot is created). Typically, delta plots in the Simon effect have a negative slope, that is, the Simon effect is especially pronounced for fast reactions and gets smaller as RT increases. On the contrary, delta plots in the Eriksen task usually have a positive slope, that is, the typical Eriksen effect gets more pronounced as RT increases (e.g., Mattler, 2003, Ulrich et al., 2015).

Differently shaped delta plots have been interpreted as the signature of different underlying processing mechanisms (e.g., Wiegand & Wascher, 2005, 2007). However, Ulrich et al., (2015) proposed a common framework to account for congruency effects and delta functions in different conflict tasks: the Diffusion Model for Conflict Tasks (DMC). The basic assumption of this model is that congruency effects arise as a consequence of two superimposed processes, a controlled diffusion process representing the accumulation of task-relevant information and an automatic one representing the processing of task-irrelevant information. The output of the latter process is assumed to be pulse-like and rather short-lived, thus reflecting that task-irrelevant information affects processing initially but then this influence decays. Importantly, the exact timing and amplitude of the automatic activation peak determine the magnitude of the congruency effect and the shape of the corresponding delta function. For example, an early peak and fast decay of the automatic activation entails that the processing conflict unfolds early during target processing, resulting in negative going delta plots, as in the typical Simon task, whereas a later peak and slower decay entails that the conflict is maximal later during target processing, resulting in positive-going delta plots as in the typical Eriksen task. Therefore, the DMC provides a plausible common processing architecture for stimulus processing in different conflict tasks.

Within this framework, behavioral adaptation in conflict tasks may be modeled through changes in the amplitude of the automatic activation peak (Ulrich et al., 2015). For instance, the automatic activation peak may be suppressed after a recent or frequent experience of conflict. Moreover, it is conceivable that such an amplitude suppression might be locked to the time course of processing, rather than the specific stimulus information represented by the automatic process. Accordingly, for example, if the irrelevant information in Eriksen trials has its maximal impact rather late during target processing, conflict adaptation (i.e., suppressing or enhancing the automatic activation) might also be confined to this late phase of processing. Consequently, the impact of such adaptation on Simon trials would be presumably small or absent, since the Simon conflict arises much earlier in stimulus processing. Even though this conception of behavioral adaptation as a time-dependent process is speculative, it might provide a new perspective on the—so far—rather inconclusive literature regarding transfer of global and local adaptation effects. Therefore, a promising novel avenue for future research on the transfer of behavioral adaptation might include the investigation of delta plots or experimental manipulations of the relative processing speed or duration of task-relevant and task-irrelevant information (cf. Dyer, 1971, Ellinghaus et al., 2018, Hommel, 1994, Hübner and Töbel, 2019, Lu & Proctor, 2001, Mattler, 2003, Mittelstädt & Miller, 2020, Simon et al., 1976).

### Conflict monitoring or contingency-based adaptation?

As outlined in the Introduction, there is an ongoing debate about whether phenomena like the Gratton and LWPC effects reflect higher-level mechanisms involving conflict detection and cognitive control or whether they are predominantly based on lower-level feature-based contingency learning (Schmidt, 2013, 2019). Our aim was not to disentangle lower-level effects such as feature repetition or binding effects from higher-order effects involving cognitive control processes based on conflict detection (Braem et al., 2019), however, so we did not control for stimulus-response repetitions/alternations or stimulus feature contingency confounds (with the exception of using diagnostic items in Experiment 2). Instead, our aim was to extend previous research on cross-task transfer of LWPC and Gratton effects to the new combination of Eriksen and Simon conflict tasks. Accordingly, we acknowledge that the present findings may, in principle, reflect either or both of these two classes of low- and high-level mechanisms. The pattern of behavioral adaptation effects observed in the present design is, in retrospect, more compatible with the ‘contingency learning’ view of behavioral adaptation: pronounced Gratton and LWPC effects within each task (where stimulus repetition and contingency confounds are present), but not across tasks were observed. Accordingly, a single, contingency-based mechanism may underlie the different types of behavioral adaptation investigated in the present study. At the same time, it seems clear that with the present design and tasks, there is no evidence for a higher-level, task-unspecific mechanism based on cognitive control (even though this leaves open the possibility that separate, more task-specific mechanisms inducing cognitive control may have been at play). Of course, it remains an open question whether a different pattern of results would emerge when stimulus-response contingencies are also controlled within each single task. In fact, it has been suggested that higher-level cognitive control mechanisms may only become engaged in the absence of other informative cues, for example, if stimulus contingencies are weak or absent (e.g., Bugg, 2014).

### Conclusions

Summarizing the present study, in two experiments Eriksen and Simon task trials were randomly intermixed. We observed both Gratton and LWPC effects which were at least as large in the Simon task as in the Eriksen task. These effects indicate that the influence of irrelevant spatial (Simon task) and identity (Eriksen task) information on the processing of task-relevant information varies along with the trial-by-trial and block-wide variations in target-distractor congruency. On a theoretical level, such local and global adaptation may be attributed to contingency-based and/or conflict-monitoring mechanisms. Both effects were shown to be task-type specific, that is, they did not transfer from one task type to the other. On the one hand, these findings add another example of task-specific adaptation to the bulk of studies investigating transfer of Gratton effects. On the other hand, regarding global adaptation, our results differ from those of two previous studies employing Simon and Stroop conflicts which demonstrated transfer of LWPC effects when the target dimension was identical for the two tasks. Therefore, our results indicate that such global effects may not (or at least not entirely) depend on enhancement of the target dimension. Other potential factors for the lack of transfer of global adaptation effects between the Simon and the Eriksen task (and vice versa) include the nature of the conflicting information in the Eriksen task, the spatial layout of stimuli, the time course of the automatic processing of conflicting information, and finally, the presence of predictive stimulus contingencies in the present design.

## Figures and Tables

**Fig. 6:**
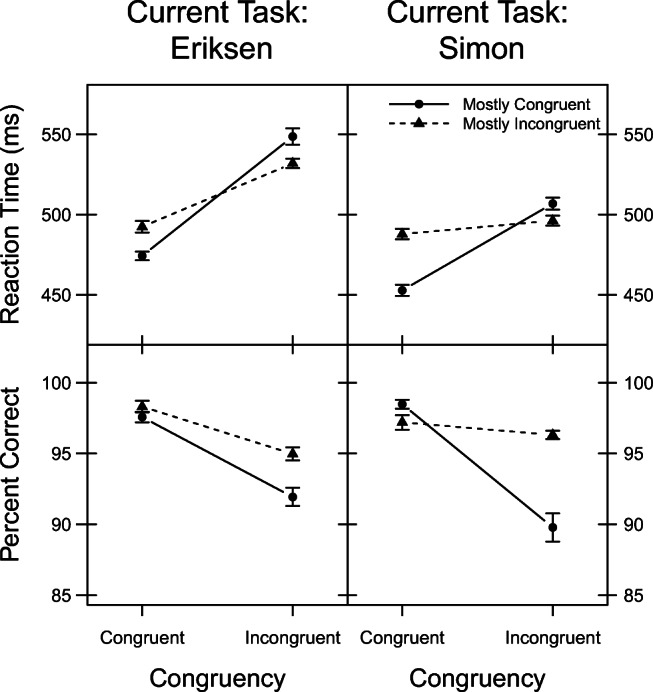
Mean reaction time (RT, in ms, *upper panels*) and mean percentage of correct responses (PC, *lower panels*) as a function of congruency, current task, and congruency proportion in Experiment 1. *Error bars* reflect ± 1 within-subjects standard error of the mean according to a suggestion of Morey (2008)

**Fig. 7:**
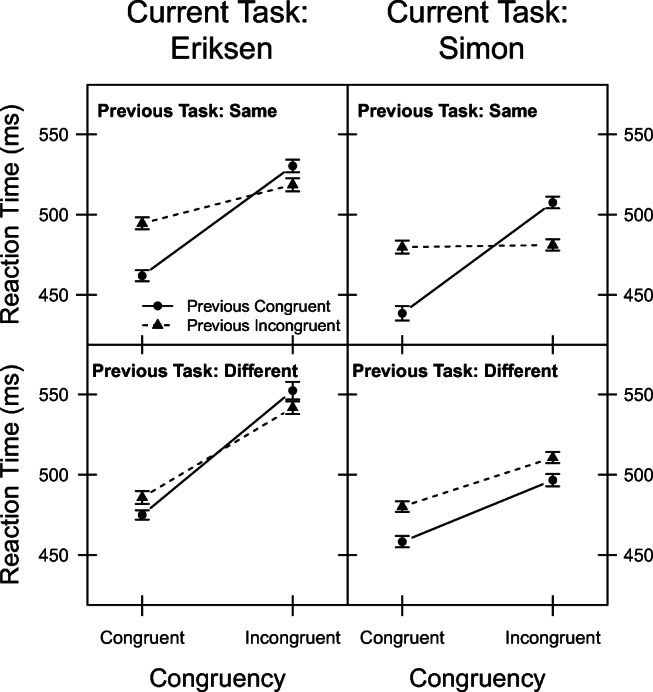
Mean reaction time (RT, in ms) as a function of congruency, current task, previous task, and previous congruency in Experiment 1. *Error bars* reflect ± 1 within-subjects standard error of the mean according to a suggestion of Morey (2008)

**Fig. 8:**
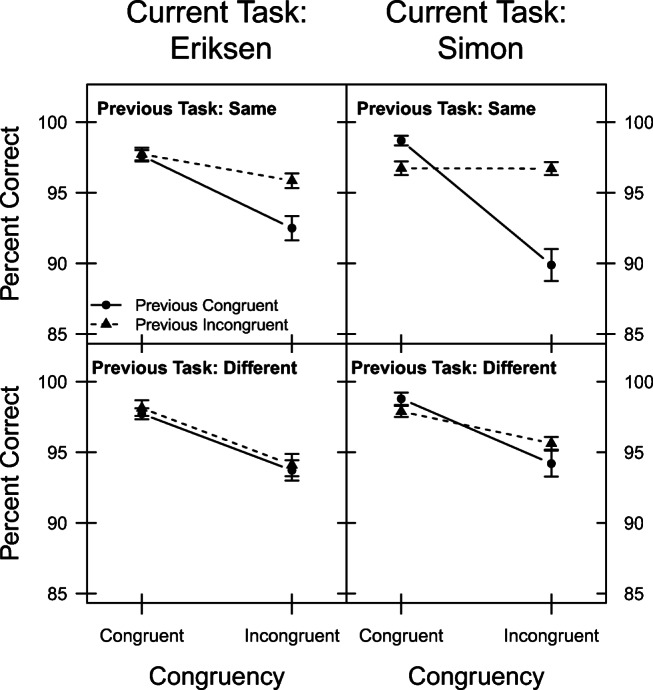
Mean percentage of correct responses (PC) as a function of congruency, current task, previous task, and previous congruency in Experiment 1. *Error bars* reflect ± 1 within-subjects standard error of the mean according to a suggestion of Morey (2008)

**Fig. 9:**
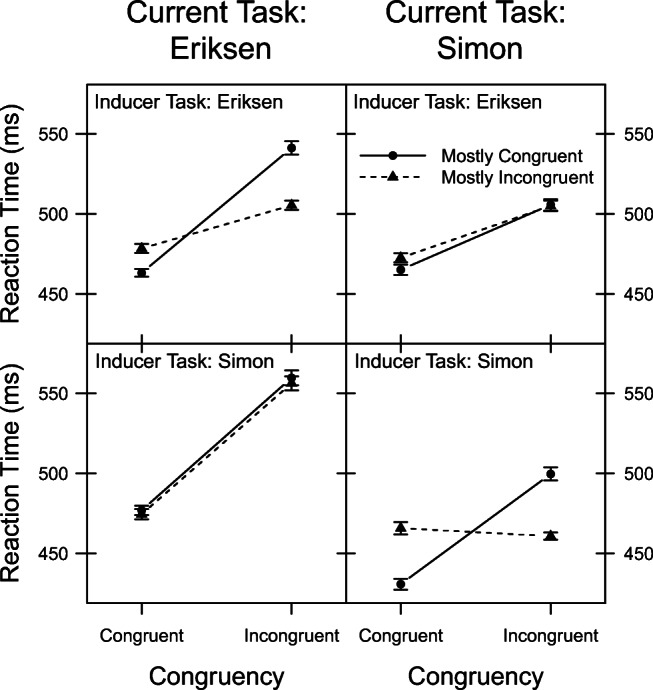
Mean reaction time (RT, in ms) as a function of congruency, current task, inducer task, and congruency proportion in Experiment 2. *Error bars* reflect ± 1 within-subjects standard error of the mean according to a suggestion of Morey (2008)

**Fig. 10:**
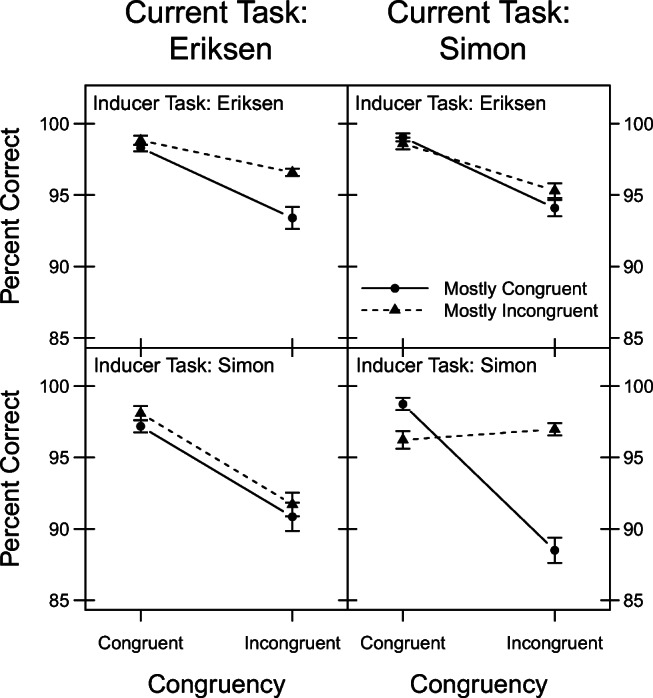
Mean percentage of correct responses (PC) as a function of congruency, current task, inducer task, and congruency proportion in Experiment 2. *Error bars* reflect ± 1 within-subjects standard error of the mean according to a suggestion of Morey (2008)

**Fig. 11:**
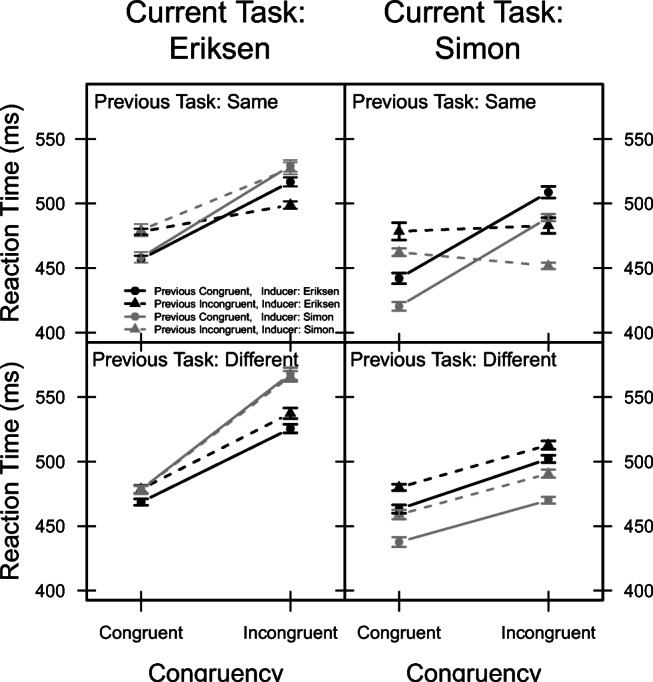
Mean reaction time (RT, in ms) as a function of congruency, current task, previous task, previous congruency, and inducer task in Experiment 2. *Error bars* reflect ± 1 within-subjects standard error of the mean according to a suggestion of Morey (2008)

**Fig. 12:**
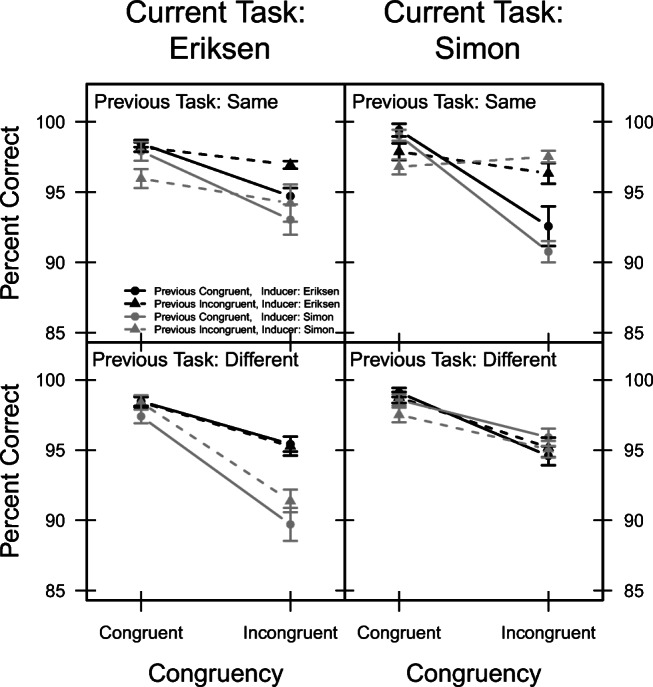
Mean percentage of correct responses (PC) as a function of congruency, current task, previous task, previous congruency, and inducer task in Experiment 2. *Error bars* reflect ± 1 within-subjects standard error of the mean according to a suggestion of Morey (2008)

## Appendix A: Mean Reaction Time and Percentage of Correct Responses

This study focuses on how congruency effects in the Eriksen and in the Simon task are modulated by within- and across-task variations of congruency proportion and congruency in the previous trial. Therefore, analyses in the main part of the present article were conducted on congruency effects, rather than on reaction time (RT) and the percentage of correct responses (PC) directly. This Appendix contains tables and figures including this additional information, to enable an investigation of congruency effects in relation to baseline RT and PC in each task.

### Experiment 1 – List-Wide Proportion Congruent Effect

**Table 2:** Mean reaction time (RT, in ms) and percentage of correct responses (PC) in congruent and incongruent trials, as well as congruency effects for RT (Δ_*R**T*_) and PC (Δ_*P**C*_), for each task (T) and congruency proportion (P) in Experiment 1.

T	P	RT			PC		
		Congruent	Incongruent	${\Delta }_{{\text {PC}}^{\text {a}}}$	Congruent	Incongruent	${\Delta }_{{\text {PC}}^{\text {b}}}$
Eriksen	MC	474 (63)	549 (83)	75	97.6 (1.9)	91.9 (6.1)	5.7
	MI	492 (72)	532 (70)	40	98.3 (2.6)	95.0 (4.6)	3.3
Simon	MC	453 (55)	507 (74)	54	98.5 (1.8)	89.8 (8.9)	8.7
	MI	488 (68)	496 (62)	8	97.2 (3.2)	96.3 (3.1)	0.9

SDs are given in parentheses.

Small deviations from the Δ values reported in the main text are due to rounding error.

^a^ Δ_RT_ = *M*_*i**n**c**o**n**g**r**u**e**n**t*_ − *M*_*c**o**n**g**r**u**e**n**t*_.

^b^ Δ_PC_ = *M*_*c**o**n**g**r**u**e**n**t*_ − *M*_*i**n**c**o**n**g**r**u**e**n**t*_.

**Table 3:** Results of two repeated-measures analyses of variance on reaction time (RT) and percentage of correct responses (PC), with factors Congruency, current task, and congruency proportion in Experiment 1

	RT			PC		
Effect	*F*	*p*	${\eta _{p}^{2}}$	*F*	*p*	${\eta _{p}^{2}}$
Congruency (C)	258.03	< .001	.85	62.59	< .001	.57
Current task (T)	79.19	< .001	.63	0.62	.436	.01
Congruency proportion (P)	3.28	.077	.07	40.82	< .001	.46
C × T	35.38	< .001	.43	0.20	.655	< .01
C × P	72.38	< .001	.61	52.32	< .001	.53
T × P	13.98	.001	.23	1.28	.264	.03
C × T × P	2.44	.125	.05	14.76	< .001	.24

*Note.* For all effects, *d**f*_*n*_ = 1 and *d**f*_*d*_ = 47.

### Experiment 1 – Gratton effect

**Table 4:** Mean reaction time (RT, in ms) and percentage of correct responses (PC) in congruent and incongruent trials, as well as congruency effects for RT (Δ_RT_) and PC (Δ_PC_), for each level of current task (T), previous task (PreT), and previous congruency (PreC) in Experiment 1

T	PreT	PreC	RT			PC		
			Congruent	Incongruent	${\Delta }_{\text {RT}^{\text {a}}}$	Congruent	Incongruent	${\Delta }_{\text {PC}^{\text {b}}}$
Eriksen	Same	Congruent	462 (60)	530 (76)	68	97.6 (2.7)	92.5 (7.6)	5.1
	Same	Incongruent	495 (71)	519 (68)	24	97.7 (3.2)	95.9 (3.4)	1.8
	Different	Congruent	475 (66)	552 (86)	77	97.7 (2.3)	93.7 (6.1)	4.0
	Different	Incongruent	486 (71)	542 (76)	56	98.1 (3.1)	94.1 (7.1)	4.0
Simon	Same	Congruent	439 (51)	508 (66)	69	98.7 (2.7)	89.9 (9.6)	8.8
	Same	Incongruent	480 (79)	481 (56)	1	96.7 (3.4)	96.7 (3.5)	0.0
	Different	Congruent	458 (59)	497 (72)	39	98.8 (1.6)	94.2 (7.6)	4.6
	Different	Incongruent	480 (61)	511 (71)	31	97.9 (2.8)	95.6 (4.4)	2.3

SDs are given in parentheses.

*Note.* Small deviations from the Δ values reported in the main text are due to rounding error.

^a^ Δ_RT_ = *M*_*i**n**c**o**n**g**r**u**e**n**t*_ − *M*_*c**o**n**g**r**u**e**n**t*_.

^b^ Δ_PC_ = *M*_*c**o**n**g**r**u**e**n**t*_ − *M*_*i**n**c**o**n**g**r**u**e**n**t*_.

**Table 5:** Results of two repeated-measures analyses of variance on reaction time (RT) and percentage of correct responses (PC), with factors congruency, current task, previous task (PreT), and previous congruency (PreC) in Experiment 1

	RT			PC		
Effect	*F*	*p*	${\eta _{p}^{2}}$	*F*	*p*	${\eta _{p}^{2}}$
Congruency (C)	263.46	< .001	.85	53.02	< .001	.53
Current task (T)	88.88	< .001	.65	0.31	.580	.01
Previous task (PreT)	29.33	< .001	.38	6.19	.016	.12
Previous congruency (PreC)	27.74	< .001	.37	12.20	.001	.21
C × T	33.44	< .001	.42	0.06	.806	<.01
C × PreT	9.94	.003	.17	0.19	.668	<.01
C × PreC	58.76	< .001	.56	35.54	< .001	.43
T × PreT	0.45	.504	.01	3.68	.061	.07
T × PreC	5.25	.026	.10	0.34	.563	.01
PreT × PreC	0.00	.979	< .01	7.24	.010	.13
C × T × PreT	9.84	.003	.17	1.86	.179	.04
C × T × PreC	0.49	.488	.01	11.77	.001	.20
C × PreT × PreC	41.32	< .001	.47	11.54	.001	.20
T × PreT × PreC	10.48	.002	.18	0.81	.372	.02
C × T × PreT × PreC	6.55	.014	.12	2.13	.151	.04

*Note.* For all effects, *d**f*_*n*_ = 1 and *d**f*_*d*_ = 47.

### Experiment 2 – List-wide proportion congruent effect

**Table 6:** Mean reaction time (RT, in ms) and percentage of correct responses (PC) in congruent and incongruent trials, as well as congruency effects for RT (Δ_RT_) and PC (Δ_PC_), for each level of current task (T), congruency proportion (P), and inducer task (I) in Experiment 2

T	P	I	RT			PC		
			Congruent	Incongruent	${\Delta }_{\text {RT}^{\text {a}}}$	Congruent	Incongruent	${\Delta }_{\text {PC}^{\text {b}}}$
Eriksen	MC	Eriksen	463 (49)	541 (64)	78	98.3 (1.2)	93.4 (6.5)	4.9
	MI		478 (51)	505 (47)	27	98.8 (1.8)	96.6 (2.5)	2.2
	MC	Simon	477 (48)	560 (56)	83	97.2 (3.1)	90.8 (8.7)	6.4
	MI		474 (50)	556 (58)	82	98.1 (2.5)	91.7 (7.8)	6.4
Simon	MC	Eriksen	465 (53)	506 (53)	41	99.0 (1.4)	94.1 (4.9)	4.9
	MI		473 (46)	505 (51)	32	98.6 (2.1)	95.3 (4.4)	3.3
	MC	Simon	431 (39)	500 (48)	69	98.7 (1.0)	88.5 (7.8)	10.2
	MI		466 (51)	461 (41)	-5	96.2 (3.9)	97.0 (2.4)	− 0.8

SDs are given in parentheses.

*Note.* Small deviations from the Δ values reported in the main text are due to rounding error.

^a^ Δ_RT_ = *M*_*i**n**c**o**n**g**r**u**e**n**t*_ − *M*_*c**o**n**g**r**u**e**n**t*_.

^b^ Δ_PC_ = *M*_*c**o**n**g**r**u**e**n**t*_ − *M*_*i**n**c**o**n**g**r**u**e**n**t*_.

**Table 7:** Results of two mixed-design analyses of variance on reaction time (RT) and percentage of correct responses (PC), with the between-SS factor inducer task, and the within-SS factors congruency, current task, and congruency proportion in Experiment 2

	RT			PC		
Effect	*F*	*p*	${\eta _{p}^{2}}$	*F*	*p*	${\eta _{p}^{2}}$
Inducer task (I)	0.03	.862	< .01	13.52	< .001	.13
Congruency (C)	847.44	< .001	.90	129.25	< .001	.58
Current task (T)	331.05	< .001	.78	1.11	.295	.01
Congruency proportion (P)	1.07	.304	.01	42.64	< .001	.31
I × C	12.99	.001	.12	4.25	.042	.04
I × T	153.86	< .001	.62	1.22	.272	.01
I × P	0.03	.858	< .01	3.02	.085	.03
C × T	126.13	< .001	.57	0.65	.422	.01
C × P	160.08	< .001	.63	64.26	< .001	.41
T × P	9.92	.002	.10	0.55	.458	.01
I × C × T	33.82	< .001	.26	2.70	.104	.03
I × C × P	2.15	.146	.02	12.12	.001	.11
I × T × P	7.41	.008	.07	17.70	< .001	.16
C × T × P	9.41	.003	.09	27.82	< .001	.23
I × C × T × P	141.44	< .001	.60	40.26	< .001	.30

*Note.* For all effects, *d**f*_*n*_ = 1 and *d**f*_*d*_ = 47.

### Experiment 2 – Gratton effect

**Table 8:** Mean reaction time (RT, in ms) and percentage of correct responses (PC) in congruent and incongruent trials, as well as congruency effects for RT (Δ_RT_) and PC (Δ_PC_), for each level of current task (T), previous task (PreT), previous congruency (PreC), and inducer task, in Experiment 2

T	PreT	PreC	RT			PC		
			Congruent	Incongruent	${\Delta }_{\text {RT}^{a}}$	Congruent	Incongruent	${\Delta }_{\text {PC}^{b}}$
*Inducer Task: Eriksen*								
Eriksen	Same	Congruent	457 (49)	517 (54)	60	98.5 (1.3)	94.7 (4.8)	3.8
	Same	Incongruent	478 (50)	499 (46)	21	98.2 (1.8)	96.9 (2.6)	1.3
	Different	Congruent	469 (53)	525 (53)	56	98.5 (2.1)	95.4 (4.4)	3.1
	Different	Incongruent	479 (54)	537 (58)	58	98.4 (2.4)	95.3 (5.4)	3.1
Simon	Same	Congruent	442 (45)	509 (59)	67	99.4 (2.3)	92.6 (10.5)	6.8
	Same	Incongruent	478 (67)	483 (56)	5	97.9 (4.1)	96.3 (6.0)	1.6
	Different	Congruent	463 (51)	502 (52)	39	99.1 (1.4)	94.6 (5.3)	4.5
	Different	Incongruent	480 (49)	513 (53)	33	98.7 (2.1)	95.2 (5.2)	3.5
*Inducer Task: Simon*								
Eriksen	Same	Congruent	458 (49)	529 (55)	71	97.9 (4.0)	93.0 (8.9)	4.9
	Same	Incongruent	479 (57)	527 (49)	48	96.0 (5.8)	94.2 (10.6)	1.8
	Different	Congruent	478 (49)	568 (58)	90	97.4 (3.4)	89.7 (10.0)	7.7
	Different	Incongruent	478 (49)	566 (58)	88	98.4 (2.6)	91.4 (7.5)	7.0
Simon	Same	Congruent	420 (40)	489 (43)	69	99.0 (1.0)	90.8 (6.5)	8.2
	Same	Incongruent	462 (44)	452 (40)	− 10	96.8 (3.1)	97.5 (2.5)	− 0.7
	Different	Congruent	438 (38)	470 (45)	32	98.6 (2.6)	95.9 (4.4)	2.7
	Different	Incongruent	459 (46)	491 (46)	32	97.5 (3.1)	95.1 (3.8)	2.4

SDs are given in parentheses.

*Note.* Small deviations from the Δ values reported in the main text are due to rounding error.

^a^ Δ_RT_ = *M*_*i**n**c**o**n**g**r**u**e**n**t*_ − *M*_*c**o**n**g**r**u**e**n**t*_.

^b^ Δ_PC_ = *M*_*c**o**n**g**r**u**e**n**t*_ − *M*_*i**n**c**o**n**g**r**u**e**n**t*_.

**Table 9:** Results of two mixed-design analyses of variance on reaction time (RT) and percentage of correct responses (PC), with the between-SS factor inducer task (I) and the within-SS factors congruency (C), current task (T), previous task (PreT), and previous congruency (PreC) in Experiment 2

	RT			PC		
Effect	*F*	*p*	${\eta _{p}^{2}}$	*F*	*p*	${\eta _{p}^{2}}$
Congruency (C)	744.01	< .001	.89	112.67	< .001	.55
Current task (T)	305.78	< .001	.76	4.93	.029	.05
Previous task (PreT)	165.52	< .001	.64	0.04	.851	< .01
Previous congruency (PreC)	31.74	< .001	.25	6.76	.011	.07
Inducer task (I)	0.21	.650	< .01	6.58	.012	.07
C × T	78.11	< .001	.45	0.43	.512	< .01
C × PreT	28.71	< .001	.23	3.40	.068	.03
C × PreC	114.68	< .001	.55	45.21	< .001	.32
C × I	8.13	.005	.08	1.19	.277	.01
T × PreT	14.65	< .001	.13	6.81	.011	.07
T × PreC	4.40	.039	.04	0.17	.677	< .01
T × I	122.58	< .001	.57	9.33	.003	.09
PreT × PreC	6.75	.011	.07	4.87	.030	.05
PreT × I	1.89	.172	.02	0.32	.574	< .01
PreC × I	0.00	.997	< .01	0.00	.946	< .01
C × T × PreT	31.06	< .001	.25	9.61	.003	.09
C × T × PreC	25.84	< .001	.22	10.79	.001	.10
C × T × I	22.04	< .001	.19	7.37	.008	.07
C × PreT × PreC	95.71	< .001	.50	35.08	< .001	.27
C × PreT × I	2.38	.126	.02	1.85	.178	.02
C × PreC × I	0.00	.972	< .01	1.86	.176	.02
T × PreT × PreC	7.10	.009	.07	7.99	.066	.08
T × PreT × I	6.00	.016	.06	1.97	.164	.02
T × PreC × I	0.72	.399	.01	0.00	.994	< .01
preT × PreC × I	0.96	.329	.01	0.12	.725	< .01
C × T × PreT × PreC	17.81	< .001	.16	8.40	.005	.08
C × T × PreT × I	1.37	.245	.01	5.45	.022	.05
C × T × PreC × I	2.10	.151	.02	0.30	.584	< .01
C × PreT × PreC × I	0.02	.876	<.01	1.98	.163	.02
T × PreT × PreC × I	7.92	.006	.08	8.67	.004	.08
C × T × PreT × PreC × I	6.03	.016	.06	2.65	.107	.03

*Note*. For all effects, *d**f*_*n*_ = 1 and *d**f*_*d*_ = 94.

## Footnotes

1 We thank an anonymous reviewer for pointing this out.
